# Increased elastic modulus of the synovial membrane in a rat ACLT model of osteoarthritis revealed by atomic force microscopy

**DOI:** 10.1590/1414-431X202010058

**Published:** 2020-10-07

**Authors:** Shouqian Dai, Ting Liang, Tadashi Fujii, Shuangjun He, Fan Zhang, Huaye Jiang, Bo Liu, Xiu Shi, Zongping Luo, Huilin Yang

**Affiliations:** 1 Orthopedic Institute, Department of Orthopaedics, The First Affiliated Hospital of Soochow University, Suzhou, Jiangsu, China; 2 Department of Emergency Medicine, The First Affiliated Hospital of Soochow University, Suzhou, Jiangsu, China; 3 Department of Orthopaedic Surgery, Kashiba Asahigaoka Hospital, Kashiba, Nara, Japan; 4 Department of Obstetrics and Gynecology, The First Affiliated Hospital of Soochow University, Suzhou, Jiangsu, China

**Keywords:** ACLT, Osteoarthritis, Synovitis, AFM, Elastic modulus, Proinflammatory cytokines

## Abstract

This study aimed to explore changes in nanoscale elastic modulus of the synovium using atomic force microscopy (AFM) in addition to investigate changes in synovial histomorphology and secretory function in osteoarthritis (OA) in a rat anterior cruciate ligament transection (ACLT) model. Sprague-Dawley rats were randomly assigned to sham control and ACLT OA groups. All right knee joints were harvested at 4, 8, or 12 weeks (W) after surgery for histological assessment of cartilage damage and synovitis in both the anterior and posterior capsules. AFM imaging and nanoscale biomechanical testing were conducted to measure the elastic modulus of the synovial collagen fibrils. Immunohistochemistry was used to visualize the expression of interleukin-1β (IL-1β), tumor necrosis factor-α (TNF-α), and matrix metalloproteinase-3 (MMP-3) in the synovium. The OA groups exhibited progressive development of disease in the cartilage and synovium. Histopathological scores of the synovium in the OA groups increased gradually. Significant differences were observed between all OA groups except for the posterior 4W group. The synovial fibril arrangement in all OA groups was significantly disordered. The synovial fibrils in all ACLT OA groups at each time point were stiffer than those in the sham controls. OA rats displayed a significantly higher expression of IL-1β and MMP3 in the anterior capsule. In summary, synovial stiffening was closely associated with joint degeneration and might be a factor contributing to synovitis and increased production of proinflammatory mediators. Our data provided insights into the role of synovitis, particularly stiffening of the synovium, in OA pathogenesis.

## Introduction

Osteoarthritis (OA) is the most common joint disease worldwide and a leading cause of pain and disability (1). Athletic injuries, especially anterior cruciate ligament rupture, can eventually lead to posttraumatic OA. Traditionally, this common arthritic disease has been characterized by marked changes in the structure and function of the articular cartilage. However, the precise etiology and pathogenesis of OA remains unclear with no drugs able to modulate the disease or therapies that can prevent or cure OA (2).

In contrast to earlier viewpoints, joint failure in OA is now perceived as being the result of a complex interactive pathological process in multiple articular tissues including the cartilage, synovium, meniscus, muscle, and cortical and subchondral bone (3). It is still debated as to which tissue structures undergo change initially as OA progresses. Increasing evidence from both experimental and clinical studies suggests that inflammation, especially synovitis, plays an important role in the pathologic process of OA (4). Many researchers have focused on the possibility of synovitis being the driving force behind the development of OA (5). In addition, histological changes of the synovium are often accompanied by increased vascularity, inflammatory molecule expression, and immune cell infiltration (6).

It has been reported that the mechanical cross-talk between cells and the extracellular matrix (ECM) controls the properties, function, and health of tissues (7). Synovial tissues in OA are no exception. There is active biomechanical interaction between cells and their microenvironment; the mechanical properties of the extracellular matrix (ECM), such as stiffness, and other mechanical parameters are able to regulate a variety of cellular processes, including cell morphology, proliferative and secretory potential, and propensity to migrate (8,9). A primary characteristic of OA is inflammation of the synovial membrane, suggesting possible changes in the local biomechanical environment as a consequence of either joint abnormality or synovial cell adaptation. The biomechanical properties of the synovium remain unexplored, particularly at the nanoscale. As changes in the articular cartilage in OA can be clearly detected at the nanoscale well before the appearance of differences in microscopic morphology (10), such variation in the biomechanical properties of synovial ECM at the nanoscale may also be an important indicator of synovitis, providing an explanation as to the onset of OA. Atomic force microscopy (AFM) provides a new method of both imaging the tissue structure and measuring its biomechanical properties at high resolution (11,12).

By considering the possible alterations in synovial biomechanical properties and their association with cartilage damage and synovial morphology and function, this study, for the first time, aimed to experimentally quantify the nanoscale elastic modulus of the synovial membrane in osteoarthritis using a rat anterior cruciate ligament transection (ACLT) model by AFM. We hypothesized that the biomechanical properties of the synovial membrane at the nanoscale undergo a change in the ACLT OA model, possibly closely related to the advancement of synovitis and degradation of cartilage, the latter directly affecting OA pathology.

## Material and Methods

### Animals

Seventy-two 12-week-old male adult Sprague Dawley (SD) rats (weighing 256±15 g) (JOINN Laboratories Inc. China) were used in this study. All animal experiments were approved by the Institutional Animal Care and Use Committee of Soochow University (China). All animals were housed in groups with a 12-h light/dark cycle and free access to tap water. Rats also had unrestricted access to standard laboratory animal chow, containing 1.15% calcium and 0.88% phosphorus.

### ACLT model and experimental design

The 72 animals were divided randomly into OA model and sham surgery groups, 36 animals in each. The animals in the experimental group and the control group were then equally divided into three groups according to different time spans (4, 8, and 12 weeks), with 12 animals for each time point. OA was induced in the right knee joint by ACLT as previously described (13). Rats were anesthetized by inhalation of 2% fluothane in oxygen/nitrous oxide. A medial parapatellar skin incision was created after shaving the leg to provide access to the arthritic space, then the patella was dislocated laterally. The anterior cruciate ligament was completely transected, as confirmed by a positive anterior drawer test. In the sham surgery group, the knee joint was merely opened, the right patella relocated, and the incision sutured. Rats were sacrificed 4, 8, or 12 weeks after surgery.

### Tissue preparation

The animals were euthanized by an excess of isoflurane (RWD Life Science Co., China) at 4, 8, or 12 weeks after the OA model had been established. Then, the entire knee joint of the right leg was surgically removed from each rat. Six joints from each group at every time point were fixed for 48 h in 4% paraformaldehyde at 4°C and then decalcified in 10% EDTA in 0.01M PBS for 1 month at 4°C. The decalcified specimens were embedded in paraffin. In general, knee OA occurs more commonly in the medial joint than in the lateral compartment (14). For histological analysis, 5-μm serial sections were prepared from the medial midcondylar region in the sagittal plain. The histological slices were stained using both routine hematoxylin and eosin (H&E) and safranin O/fast green to reveal histological changes in the articular cartilage and synovium.

The remaining six undecalcified joints were sliced into 20-μm serial frozen sections in the same anatomical fashion as the paraffin sections for evaluation of the biomechanical properties of the synovial membrane using AFM.

### Histological analysis

For routine histology, morphological changes in the articular cartilage and synovium were analyzed by standard H&E staining in a single section. Safranin O/fast green-stained sections were used to assess the cartilage histopathology score in accordance with the OARSI OA Cartilage Histopathology Assessment System (15).

Cartilage degeneration was assessed simultaneously using the Mankin and OARSI scoring systems. The Mankin score was calculated based on the structure of the cartilage, cells, safranin-O-staining, and tidemark integrity using a score in the range 0-14 (best to worst) (16). For the OARSI scoring system, cartilage degeneration was scored as none to severe (numerical values 0-5), as described previously (15).

Synovitis in OA is not a diffuse process and its distribution is confined to areas close to sites of chondropathy (17). As morphological changes in the synovium and severity of synovitis in the anterior capsules may be different from that in the posterior part of the sagittal sections, changes in synovial histopathology of the anterior and posterior compartments were compared in the different groups. Synovitis was examined using the validated grading system (18), including analysis of four parameters: hyperplasia of the synovial lining cell layer, activation of resident cells or synovial stroma, and inflammatory infiltration, with scores ranging 0-9 (slight to severe).

### AFM scanning and nanoindentation testing

An AFM scanner (Dimension ICON, Bruker, USA) was used at room temperature for imaging and mechanical testing. Since the properties of the collagen fibrils of the synovium could vary at different locations within the capsule, subdivisions in the synovium in the anterior and posterior capsule were measured independently for each section. Twenty collagen fibrils from each site were tested. AFM imaging and nano-mechanical testing were both performed at a scanning rate of 1 Hz using a ScanAsyst-Air probe with a curvature radius of 5 nm and a force constant of 0.4 N/m (Bruker, USA).

The diameter and D-periodic banding patterns of the collagen fibrils were recorded in AFM imaging experiments. A larger area (5×5 μm) of each sample was first scanned prior to a smaller region (1×1 μm) being re-scanned at a higher resolution (19). Statistical measurements of collagen fibril diameter and D-periodic banding were calculated by line section analysis using NanoScope Analysis Software (Bruker, USA).

The compressive elastic modulus of the collagen fibrils was measured in AFM PeakForce Quantitative Nano Mechanics (QNM) testing mode. Since the elastic modulus of the fibrils where they overlapped or regions where there were gaps differs significantly (20), only the overlapping regions were selected for measurement in this study. After calibration of the force constant k and curvature radius R of the probe, the elastic modulus was calculated using the Hertz model in the Equation, the most commonly used in AFM indentation testing of collagen fibrils (21):

$$F=\frac{4}{3}\frac{e}{\left(\text{1}–v^{\text{2}}\right)}\sqrt{R}\delta^{\frac{3}{2}}$$

where F is the indentation force, e is Young's modulus, v is Poisson's ratio, R is radius of the indenter (tip), and δ is indentation.

### Immunohistochemical analysis

Immunohistochemical analysis was conducted on decalcified sections of the right knee joint under standard procedures (22). Interleukin-1β (IL-1β), tumor necrosis factor-α (TNF-α), and matrix metalloproteinase-3 (MMP-3) in the synovium were semi-quantitatively analyzed. Sections were incubated with rabbit primary antibodies (IL-1β, dilution 1:100; TNF-α, dilution 1:100; MMP-3, dilution 1:200; all from Abcam, England) or control rabbit IgG (1:100 in 5% BSA) and goat anti-rabbit secondary antibody (Cell Signaling Technologies, USA, dilution 1:400). Slides were imaged using an Olympus light microscope (Japan) at 40× magnification. Immunohistochemical staining results were analyzed using a semi-quantitative method, as previously described (22). Each slice was observed in ten vision fields. The number of stained positive cells and their staining intensity was used for scoring, and the two scores (range: 0-12) were multiplied to obtain protein expression intensity. All sections were analyzed by two independent observers blinded to the experimental details. There was no significant difference within intra-observer and inter-observer measurements.

### Statistical analysis

Experimental data are reported as means±SD. For immunohistochemical staining analysis, differences within groups were assessed using one-way ANOVA after verification of normality. *Post hoc* comparisons between groups were tested using a least significant difference (LSD) method. For the remaining data, a comparison between the Sham and ACLT OA groups was conducted using an independent-samples *t*-test. Statistical analysis was performed using SPSS v17.0 software (IBM, USA). P values <0.05 were considered statistically significant.

## Results

### H&E staining of cartilage

**Figure 1:**
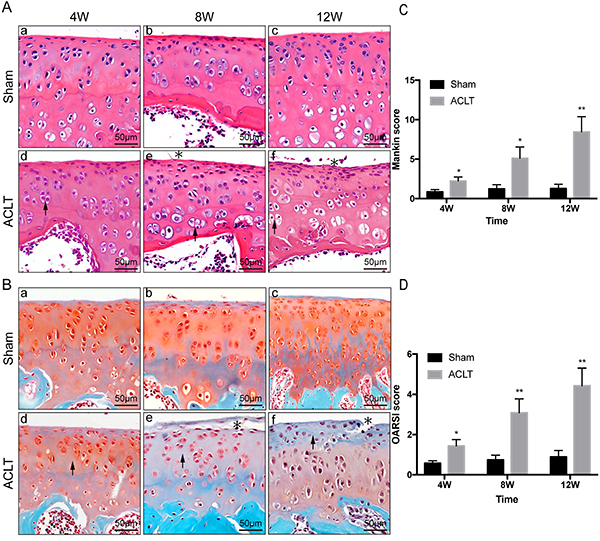
Articular cartilage degeneration in sham controls and osteoarthritic knees 4, 8, and 12 weeks (W) after anterior cruciate ligament transection (ACLT). **A**, H&E staining and (**B**) safranin O/fast green staining of cartilage in osteoarthritis groups and their respective sham controls. In all sham groups, a normal and physiological cartilage structure was observed, including a flat joint surface, natural cellular morphology, and layering with a normal tidal line. Arrows indicate the occurrence of chondrocyte clusters and an increase in cell number (**A**) and gradually declined staining intensity (**B**). Asterisks indicate cracks on the cartilage surface and a thinning of the hyaline cartilage layer (scale bar 50 μm). **C**, Mankin and (**D**) OARSI scoring were used to semi-quantitatively analyze degeneration of the cartilage. Data are reported as means±SD. *P<0.05, **P<0.01 compared to Sham (independent-samples *t*-test).

The results of H&E staining of the knee joints are shown in Figure 1. In all sham groups, a normal and physiological cartilage structure was observed, including a flat joint surface, natural cellular morphology, and layering with a normal tidal line, as shown in Figure 1Aa-c. However, the femoral condyles in the OA groups displayed a progressive pathological process in the cartilage in the longer time periods. It was clear that the 4W, 8W, and 12W ACLT OA groups represented early, middle, and late stages of OA, respectively. In the 4W group, the cartilage surface was quite smooth and flat with no visible ruptures. The only obvious change was the occurrence of chondrocyte clusters and an increase in cell number (Figure 1Ad). The cartilage surface in the 8W group was rough with minor cracks. There was an elevated cell number, density, and frequency of cell clusters, in addition to slightly disorganized tidal lines with small breaks, as shown in Figure 1Ae. The 12W ALCT OA group demonstrated features of late stage OA, including a greater number of deep cracks on the cartilage surface, indistinguishable cell layer, overlapping or missing tidal lines, and a thinning of the hyaline cartilage layer (Figure 1Af).

### Safranin O-fast green staining of cartilage

Safranin O-fast green staining can be indicative of the degree of cartilage degeneration in OA pathology. Safranin O-stained proteoglycan was visible within the cartilage matrix of all sham controls (Figure 1B). However, staining intensity gradually declined in the ACLT OA groups as OA worsened. The lightest staining occurred in the 12W ACLT OA group, where a number of regions were almost grey, implying a severe loss of proteoglycan. Both Mankin and OARSI scorings increased with longer experimental duration, all three ACLT OA groups demonstrated significant differences compared with the sham group (Mankin score: P=0.043, 0.029, 0.005; OARSI score: P=0.031, 0.009, 0.004) (Figure 1C and D).

### HE staining and histological scoring of the synovium

**Figure 2:**
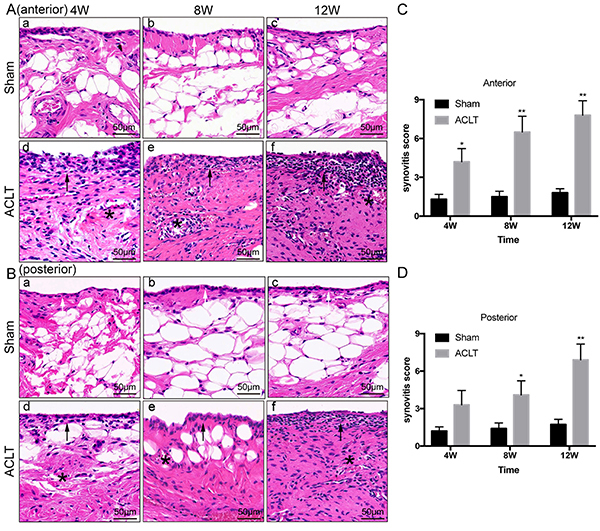
Synovial inflammation in sham controls and in osteoarthritic knees 4, 8, and 12 weeks (W) after anterior cruciate ligament transection (ACLT). Routine H&E staining of synovium in osteoarthritis groups and their respective sham controls in the (**A**) anterior and (**B**) posterior part of knee joints (scale bar 50 μm), and their corresponding histopathological scores (**C** and **D**). The lining of the tissue in the sham groups was 1-2 cells thick without hypertrophy, hyperplasia, or matrix fibrosis (white arrow). Synoviocyte hypertrophy and hyperplasia were observed with disordered tissue arrangement (black arrow), in addition to matrix edema and infiltration of inflammatory factors and cells (asterisks). Data are reported as means±SD *P<0.05, **P<0.01 compared to Sham (independent-samples *t*-test).

Although histopathologic changes in the cartilage were observed, as described above, synovial histomorphology was also altered as OA progressed. The synovial membrane in the sham groups consisted only of synoviocytes and was free of basement membrane. The lining of the tissue in the sham groups was 1-2 cells thick without hypertrophy, hyperplasia, or matrix fibrosis (Figure 2A and B). In the 4W ACLT OA group, although only lightly degraded cartilage was observed, histopathological changes to the inflamed synovium were already quite apparent. Synoviocyte hypertrophy and hyperplasia were observed with disordered tissue arrangement, in addition to matrix edema and infiltration of inflammatory factors and cells (Figure 2Ad). In the 8W group, histopathologic changes to the synovium, as described above, increased, with interstitial fibrosis and clearly apparent vascular proliferation (Figure 2Ae). Twelve weeks post-surgery, the synoviocytes of the lining had proliferated to more than ten layers, combined with significant matrix fibrosis (Figure 2Af).

Synovitis histological scoring showed significant differences between all OA groups except for the posterior 4W group (P=0.110) and the corresponding sham controls (Figure 2C and D). As time after surgery increased, histopathological scoring of the synovium in the ACLT OA group increased gradually in both the anterior and posterior capsules.

### Changes to nano-biomechanical characteristics of the synovium using AFM

**Figure 3:**
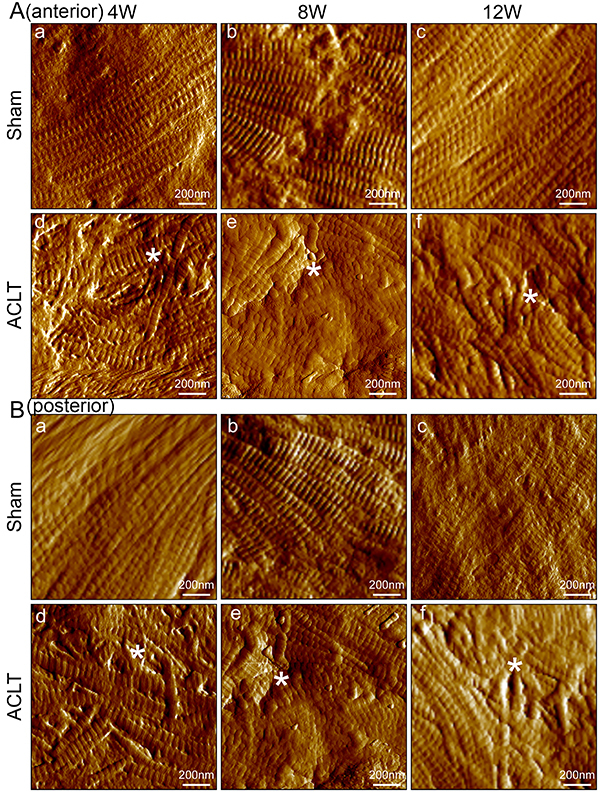
Representative atomic force microscopy images of synovial collagen fibrils in sham controls and osteoarthritic knees 4, 8, and 12 weeks (W) after anterior cruciate ligament transection (ACLT). Knees were scanned (**A**) anteriorly and (**B**) posteriorly, separately (scale bar 200 nm). Compared to the sham controls, the arrangement of synovial fibrils in all osteoarthritis groups appeared significantly disordered in both anterior and posterior capsules (asterisks). However, there were no significant differences in the diameter and D-periodic banding patterns between the sham controls and osteoarthritis groups.

In order to exam the nano-biomechanical properties of the synovial membrane, AFM imaging and biomechanical testing at the nanoscale was conducted. Representative AFM images of the anterior and posterior synovium of the knee joint from the sham controls and ACLT OA groups are shown in Figure 3. Fibril diameter, arrangement, and D-periodic banding patterns are visible in the images. Compared to sham controls, synovial fibrils in all ACLT OA groups were significantly disordered in both the anterior (Figure 3A) and posterior (Figure 3B) capsules. However, there were no significant differences in the diameter and D-periodic banding patterns between the sham controls and ACLT OA groups (P>0.05). The maximum and minimum fibril diameter in all groups was 133.62±21.03 nm *vs* 133.03±10.27 nm (P=0.956). The maximum and minimum D-periodic banding in all groups was 68.19±12.73 nm *vs* 65.43±2.57 nm (P=0.647).

**Figure 4:**
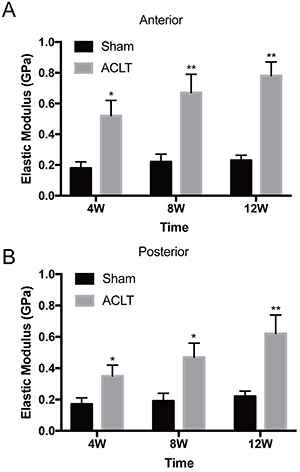
Mean elastic moduli of synovial collagen fibrils in sham controls and osteoarthritic knees 4, 8, and 12 weeks (W) after anterior cruciate ligament transection (ACLT). The elastic modulus was measured both in the (**A**) anterior and (**B**) posterior joint capsules of all animals. Compared with the sham control group (0.18±0.04 GPa at 4W, 0.22±0.05 GPa at 8W, 0.23±0.03 GPa at 12W), fibrils stiffened in the anterior joint capsule at each time point (0.52±0.10 GPa at 4W, 0.67±0.12 GPa at 8W, 0.78±0.09 GPa at 12W). In the posterior joint capsule, this change was significant (0.35±0.07 *vs* 0.17±0.04 GPa at 4W, 0.47±0.09 *vs* 0.19±0.05 GPa at 8W, 0.62±0.12 *vs* 0.22±0.03GPa at 12W). Data are reported as means±SD. *P<0.05, **P<0.01 compared to Sham (independent-samples *t*-test).

The elastic moduli of synovial collagen fibrils at the nanoscale are presented in Figure 4. For both anterior (Figure 4A) and posterior (Figure 4B) capsules, the synovial fibrils in the ACLT OA groups at all time points were stiffer than those in the sham controls (anterior: P=0.010, 0.006, <0.001; posterior: P=0.049, 0.022, 0.009). The elastic modulus of the synovium 4W post-surgery was significantly higher in the anterior and posterior capsules, 2.89- and 2.06-fold greater than those of the corresponding sham group. The modulus of the synovium increased further at 8 and 12 weeks, to 3.05- and 3.39-fold higher in the anterior and 2.47- and 2.82-fold higher in the posterior capsules, respectively, compared with the sham controls. The elastic modulus of the sham synovium, not surprisingly, did not change significantly throughout the study.

### Expression levels of IL-1&mac_bgr;, TNF-&mac_agr;, and MMP-3 in the synovium revealed by immunohistochemical staining

**Figure 5:**
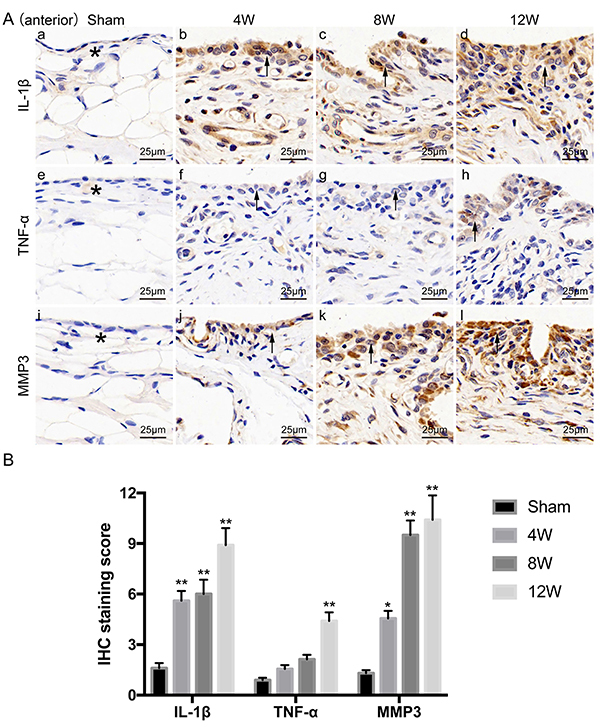
Expression levels of interleukin (IL)-1β, tumor necrosis factor (TNF)-α, and matrix metalloproteinase-3 (MMP-3) in the synovium of sham controls and osteoarthritic knees 4, 8, and 12 weeks (W) after anterior cruciate ligament transection (ACLT). Immunohistochemical staining of IL-1β (**Aa**-**d**), TNF-α (**Ae**-**h**), and MMP-3 (**Ai**-**l**) expressed in the synovium of the anterior capsule of sham controls and osteoarthritis groups (scale bar 25 μm), and (**B**) their semi-quantitative analysis. In the sham controls, light yellow-stained IL-1β, TNF-α, and MMP3 were found in the cytoplasm and principally distributed within the extracellular matrix of the synovium (*). In the ACLT groups, however, these proteins were predominantly distributed in the synovial cytoplasm of the lining layer and sub-lining layer, with brown-stained particles (black arrow). Data are reported as means±SD. *P<0.05, **P<0.01 compared to Sham (ANOVA).

The expressions of IL-1β, TNF-α, and MMP-3 in the synovium are shown in Figure 5. Specifically, ACLT OA rats at all time points displayed significantly higher levels of IL-1β and MMP-3 as experimental time increased, compared with the sham controls (IL-1β: P=0.004, 0.002, <0.001; MMP-3: P=0.048, <0.001, <0.001). However, expression levels of TNF-α were relatively lower, with no significant changes observed in the 4W and 8W groups compared with sham controls (P=0.397, 0.051). Only 12 weeks after surgery, there was a significant difference in TNF-α expression between the sham control and OA groups (P=0.001). In the sham controls, light yellow-stained IL-1β, TNF-α, and MMP3 were found in the cytoplasm and principally distributed within the extracellular matrix of the synovium. In the ACLT OA groups, however, these proteins were predominantly distributed in the synovial cytoplasm of the lining layer and sub-lining layer, with brown-stained particles. Such staining was also observed in inflammatory cells, such as monocytes and macrophages. The synovial extracellular matrix in the OA groups displayed lighter staining compared with cytoplasmic staining, but heavier than that of the sham controls.

## Discussion

A large number of researchers have recently focused on the possibility that synovial inflammation is an important factor in the pathology and progression of OA (5). To date, however, the majority of studies have only observed macroscale and microscale morphological changes in the synovium in OA, and to our knowledge, no study has explored its nanoscale morphology and biomechanical properties, the foundation of macroscopic changes. In this study, we assessed OA-related histopathological changes in the cartilage and synovium in an ACLT model of the rat knee, with particular attention to alterations of nanoscale biomechanical properties of the synovium, hypothesized to be closely related with synovitis and progression of OA.

The ACLT model has been shown to be an important tool in OA pathological research. The results of this study demonstrated that cartilage degeneration gradually increased with time in an ACLT OA model. These results agree with previous studies (23), suggesting that OA is a progressive and time-dependent disease.

In addition to cartilage damage, synovial inflammation is another important hallmark of OA. Inflammation, especially synovitis, is believed to play a substantial role in the onset of OA and its progression. A number of studies have found that synovial inflammation occurs earlier than cartilage damage in mild OA (24,25). It has also been reported that synovitis is closely related to higher levels of degeneration in articular cartilage in human OA and can indicate progression of arthritic damage (16). In the present study, varying degrees of synovitis were observed in the different ACLT OA groups, in addition to cartilage damage. After 4 weeks of induction of OA, synovitis in the medial joint compartment was significantly greater than it was in the sham controls, and greater still in the 8-12W groups. Similar results have been reported previously, chondral defects and associated synovitis being closely associated in the medial tibiofemoral compartment of the knee (17) with synovitis directly implicated in the initiation and progression of OA. In addition, although pathological changes to the synovium were apparent in advanced OA (12 weeks after surgery), a significant increase in synovial hypertrophy and inflammation was observed as early as 4 weeks after induction of OA, while changes in the cartilage were mild or at an early stage. This could be attributed to the possible early contribution of synovitis to the pathogenesis of OA. Interestingly, the present study also demonstrated that the histopathological features of synovitis in the anterior part of knee capsule were more serious than within the posterior part. This suggests that histopathological changes in the inflammatory synovium can vary in different locations of the joint. However, it remains unclear whether synovitis is the primary cause or whether it is the result of cartilage degeneration and deserves further research.

Past studies of the pathophysiology of synovitis in OA have primarily concentrated on synovial histomorphological alterations at the macro and micro level. Few studies have discussed changes to nanoscale biomechanical properties of the synovium in models of OA. Our data revealed, for the first time, that the elastic modulus of synovial collagen fibrils in all OA groups increased significantly in both anterior and posterior capsules compared with the corresponding sham controls. However, the diameter and D-periodic banding patterns of the collagen fibrils were similar in both the ACLT OA and sham groups at all time points. In other words, the synovial ECM in all experimental groups stiffened dramatically after ACLT surgery. Furthermore, fibrotic disorders were also found in the OA groups except for increased synovial stiffness, which can be considered a form of fibrosis, as the process involves deposition of a dense, disorganized ECM of collagen (26).

It is well-known that the ECM is a physiochemical structure that can regulate the three-dimensional alignment and behavior of cells in specific tissues. Their unique structure, physical properties such as matrix stiffness, and biochemical constitution of the ECM significantly influence matrix-cellular communications (27). It was reported by EI-Mohri et al. (8) that elevated matrix stiffness resulted in increased rates of fibroblast proliferation and fiber formation. In addition, variations in stiffness of the ECM can stimulate different cellular morphologies and increase key inflammatory mediators (28). Therefore, changes in the proliferation of synoviocytes (a type of fibroblast) and their functioning in addition to progression of synovitis are possibly associated with alteration in synovial matrix stiffness. This may at least partly explain the characterization of synovitis including hypertrophy, hyperplasia of synovial lining cells, and ECM fibrosis in OA. Further investigation is required to clarify the precise association between the stiffening of the ECM within the synovium and synovitis in OA knees and whether synovial stiffness reflects the level of disease or dysfunction.

It has been reported that a number of proinflammatory mediators can be released by bone, cartilage, and the synovium (29,30). Synovitis can be sustained by cytokines such as IL-1β and TNF-α, which can further promote the production of MMPs and other proteinases (31,32). Such cytokines produced by an inflammatory synovium, including IL and TNF, are able to induce a degradative cascade leading to joint damage (33). A previous study linked the activation of synovial inflammatory cells to damage in cartilage in a murine model of OA (34), in which proinflammatory cytokines were thought to be mediators (35). Our data indicated that expressions of IL-1β, TNF-α, and MMP-3 proteins in the OA synovium were clearly elevated in the ACLT groups at all time points. Positive staining (corresponding to protein expression levels) appeared to correlate with infiltration of inflammatory cells into the synovium. These proteins were expressed at the highest levels in cells of the synovial lining. This distribution of IL-1β, TNF-α, and MMP-3 expression in the synovium is in agreement with the rationale that bioactive cytokines and proteoenzymes can be readily secreted into the synovial fluid and then dispersed into cartilage to induce cartilage damage (36). This, at least partly, supports the observation that OA pathology and synovitis in the 12W group were more serious than found in the 4W and 8W groups.

MMP is a zinc-dependent protease that has a crucial role in ECM decomposition process in pathologies such as arthritis and tumor invasion (37). MMP-3 is a member of the MMP family and has been shown to be involved in a variety of inflammatory and oncological conditions. It was reported that MMP-3 is highly expressed in the synovial cells of osteoarthritis and can promote the proliferation of synovial cells and inhibit apoptosis (38). In addition, Chen et al. (39) found that reduction of MMP-3 mRNA and protein expression can protect the cartilage and treat osteoarthritis. In our study, MMP-3 was found to be highly expressed in synovial cells of osteoarthritis in all OA groups. Based on previous research and our data, we inferred that high expression of MMP-3 was closely related to the occurrence and development of synovitis and osteoarthritis.

There are a number of potential limitations of this study. Firstly, the pathophysiology and pathogenesis of ACLT OA is complicated and unclear. Although the onset of synovitis and synovial stiffening in OA in addition to cartilage degradation may be due to ongoing arthritic instability, the results only suggest that synovitis and synovium stiffening is closely involved in the development of OA. We cannot absolutely determine which represents the initiator of the disease and whether stiffening of the synovium and synovitis promote the progression of OA. Secondly, ACLT-induced models of biomechanical instability represent posttraumatic OA with arthritic instability, and the validity of direct translation of ACLT models still require careful appraisal in future studies.

In the present study, we demonstrated in a rat OA model that the elastic modulus of synovial collagen fibrils in ECM at the nanoscale increased significantly and that synovitis, especially stiffening of the synovium, was closely related to joint degeneration in knee OA, particularly in the anterior arthritic capsule. Our data provided insights into the role of synovitis, particularly stiffening of the synovium, in OA pathogenesis.
